# Pro-Dopamine Regulator (KB220) A Fifty Year Sojourn to Combat Reward Deficiency Syndrome (RDS): Evidence Based Bibliography (Annotated)

**Published:** 2018-12-04

**Authors:** Blum Kenneth, Modestino J. Edward, Gondre Lewis C. Marjorie, Baron David, Steinberg Bruce, Thanos K. Panayotis, Downs B. William, Siwicki Davis, Lott Lisa, Braverman R. Eric, Moran Mark, Miller David, Fried Lyle, Badgaiyan D. Rajendra

**Affiliations:** 1 Western University Health Sciences, Graduate School of Biomedical Sciences, Pomona, CA; 2 Eotvos Loránd University, Institute of Psychology, Budapest, Hungary; 3 Department of Psychiatry, Wright State University Boonshoft School of Medicine and Dayton VA Medical Center, Dayton, OH, USA; 4 Department of Clinical Neurology, Path Foundation NY, New York, USA; 5 Department of Psychiatry, University of Vermont, Burlington, VM, USA; 6 Dominion Diagnostics, LLC., North Kingston, RI, USA; 7 Division of Precision Addiction Management, Geneus Health, LLC., San Antonio, TX, USA; 8 Division of Addiction Therapy Research, Nupathways, Inc., Meadow Ridge Dr. Innsbrook, MO, USA; 9 Division of Neurogenetic Research & Addiction Therapy, The Florida House Experience, Deerfield Beach, FL, USA; 10. Division of Nutrigenomics, Victory Nutrition International. LLC., Lederach, PA; 11 Department of Psychology, Curry College, Milton, MA, USA; 12 Departments of Anatomy & Psychiatry & Behavioral Sciences, Howard University School of Medicine, Washington, DC, USA; 13 Behavioral Neuropharmacology & Neuroimaging Laboratory on Addiction, Research Institute on Addictions, University of Buffalo, Buffalo, NY, USA; 14 Department of Psychiatry, Ichan School of Medicine at Mount Sinai, New York, NY, USA

**Keywords:** Annotated, Kb220, Restoregen Variant, Genetic Addiction Risk Score (GARS®), Precision Addiction Management (PAM®)

## Abstract

**Background:**

We are facing a significant challenge in combatting the current opioid and drug epidemic worldwide. In the USA, although there has been notable progress, in 2017 alone 72,000 people died from a narcotic overdose. The NIAAA & NIDA continue to struggle with innovation to curb or eliminate this unwanted epidemic. The current FDA list of approved Medication Assistance Treatments (MATS) work by primarily blocking dopamine function and release at the pre-neuron in the nucleus accumbens. We oppose this option in the long term tertiary treatment but agree for short term harm reduction potential.

**Bibliography Presentation:**

As an alternative motif, the utilization of a well-researched neuro-nutrient called KB220 has been intensely investigated in at least 38 studies showing evident effects related to everything from AMA rate, attenuation of craving behavior, reward system activation including BOLD dopamine signaling, relapse prevention, as well as reduction in stress, anger, and aggressive behaviors. We are continuing research especially as it relates to genetic risk, including the now patented Genetic Addiction Risk Score (GARS^®^) and the development of “Precision Addiction Management (PAM)” to potentially combat the opioid/psychostimulant epidemic.

**Conclusion:**

Based on animal research and clinical trials as presented herein, the Pro-Dopamine Regulator known as KB220 shows promise in the addiction and pain space. Other neurobiological and genetic studies are required to help understand the mechanism of action of this neuro-nutrient. However, the evidence to date points to induction of “dopamine homeostasis”enabling an asymptotic approach for epigenetic induced “normalization” of brain neurotransmitter signaling and associated improved function in the face of either genetic or epigenetic impairment of the Brain Reward Cascade (BRC).

With that said, we are encouraged about these results as published over the last 50 years and look forward to continued advancements related to appropriate nutrigenomic solutions to the millions of victims of all addictions (from drugs to food to smoking to gambling and gaming especially in our next generation) called Reward Surfeit Syndrome (RSS) in adolescents and Reward Deficiency Syndrome (RDS) in adulthood.

## Summary

Such phenotypes are precisely characterized using the Genetic Addiction Risk Score (GARS). Dopamine homeostasis may thus be achieved via PAM, the customization of neuro-nutrient supplementation (KB220 variants based on previous research with KB220) tied to the GARS test result, along with a behavioral/ spiritual intervention [41,42].

## Introduction

The opioid crisis/epidemic including over-prescription and overuse of opioid drugs in North America since over the last three decades is of major concern. Opioids fit within a diverse class of moderately strong painkillers, including oxycodone, hydrocodone, and a powerful painkiller, fentanyl, which is synthesized to resemble other opiates such as opium-derived morphine and heroin. The CDC estimate of $78.5 billion as the annual cost of prescription opioid abuse is only a fraction of the total cost of chronic pain on society. Using data from 2008, researchers from Johns Hopkins University estimated that the economic cost of pain in the United States ranged from $560 to $635 billion annually. Others have estimated that it is now north of one trillion. The origins of the opioid crisis, which killed approximately 72k last year based on federal figures, were also shown in 1986 when the World Health Organization identified the inadequate treatment for pain as a serious public health concern and encouraged physicians to prescribe opioid analgesics for cancer patients. Drug overdose deaths are continuing to increase in the US:

- Within the years of 1999 to 2016, greater than 630k people passed away from an overdose.
- Approximately 66% of the more than 63.6k overdose fatalities in 2016 involved an opioid.
- In 2016, the number of overdose deaths involving opioids (including prescription opioids and illegal opioids, including heroin and illicitly manufactured fentanyl) was five times higher than in 1999.
- On average, 115 Americans die every day from an opioid overdose (see Figure 1).

While the current use of MATS may have the ability to reduce societal harm in the short term, they are only the tip of the iceberg and do not address the cause of the epidemic. We contend that instead of blocking brain dopamine, balancing dopamine, is more reasonable.

In this regard, we present here in an annotated body of work representing approximately five decades of research related to a neuro-nutrient called KB220, providing a scientific basis for its adoption in the addiction and even pain space.

## Bibliography Presentation

- In genetically prone alcohol-liking mice, the increase of brain opioid peptides, such as enkephalin, by administering D-phenylalanine (now in KB220/restoregen), significantly reduced their intake of alcohol to the same level of alcohol-hating mice [1]. This represents the first pharmacogenetic engineering experiment in the addiction space.
- In two independent studies in mice, it has been shown that both the serotonin precursor amino-acid L-Tryptophan, and the dopamine precursor L-Dopa, significantly increase sleep-time in mice following alcohol administration compared to saline placebo [2,3]. This experiment represents the possible commonality of neurotransmitter involvement in alcohol induced withdrawal symptomatology and further support for the Brain Reward Cascade (BRC) concept.
- Administering the KB220 variant in 22 sever alcoholics attending a residential program undergoing withdrawal resulted in an attenuated drink score, required no PRN benzodiazepines, and patients ceased having tremors 24 hours earlier and had less depression [4]. This provides the first indication that Prodopamine regulation could be helpful during detoxification from alcohol.
- In a randomized placebo-controlled study of 62 severe poly-drug abusers attending residential treatment, the KB220 variant administered at intake resulted in: reduced stress as measured by skin conductance, improved Physical and BESS (behavioral, emotional, social and spiritual) Scores, and a six-fold decrease in leaving Against Medical Advice (AMA) rates [5]. In this double-blinded placebo controlled study evidence is presented whereby strees reduction and AMA rate is a function of dysregulation of neurotransmitter balance.
- A study involving 54 cocaine-dependent mixed gender patients over a 30-day period in residential treatment included three groups: administration of KB220 specific formula for psychostimulant abuse called Tropamine (T); administration of KB220 specific formula for alcohol abuse called SAAVE(S); and no supplement [C]. Results demonstrate the AMA rates to be:- [C] 37.5%; [S] 26.6%; [T] 4.2%. Moreover, Tropamine decreased the AMA rate by significant reduction of drug hunger. This represents the beginning of precision medication therapy [6]. While the basic formula relating to Pro-dopamine regulation is tantamount to neurotransmitter dysfunction it does suggest that specific formulae in terms of drug of choice seems more prudent as a treatment option.
- In an out-patient DUI treatment program involving 60 alcoholics and cocaine dependent people, the administration of SAAVE for alcoholics and Tropamine for Cocaine resulted in reduced relapse rates and enhanced recovery in a 10-week outpatient setting. Specifically, after ten months, the recovery rate when SAAVE was administered for alcoholics was 73% (26% relapse). Furthermore, the recovery rate when Tropamine was administered for cocaine addicts was 53% (47% relapse rate). The average relapse rate for both alcoholism and cocaine dependence ranges from 87–93% [7]. Certainty, this finding has relevance to the current drug court dogma pointing to induction of dopamine homeostasis as being important for longterm recovery.
- In an outpatient bariatric clinic, 27 very serious carbohydrate bingers received a KB220 variant called PhenCal 103 over a 90-day program, whereby 16 were administered PhenCal 103 and 11 were not. The results indicated that the average weight loss to PCAL-103 was 26.96 lbs. vs. 10.2 in the control group. Relapse was 18.2% in the PCAL-103 group vs. 81.8% in the control group [8]. The importance of this study is to support common mechanisms related to Reward Deficiency Syndrome (RDS) including Obesity as a sub classification of RDS.
- In a state psychiatric hospital, a total of 12 severe cocaine-dependent patients underwent a randomized, double-blind placebo-controlled experiment involving a KB220 variant similar to Tropamine, whereby eight patients were placed on KB220 variant and four on placebo. The results showed that cocaine craving decreased significantly in KB220 compared to the placebo group [9]. This experiment although a small number supports the role of dopamine regulation in cocaine craving.
- In an open trial of 20 healthy non-addicted males, cognitive processing speeds in healthy young adult volunteers were measured pre and post 28–30 days of supplementation with a KB220 variant. Cognitive processing speeds were shown to be enhanced by a statistically significant amplitude of the P300 component of the Event-Related Potentials (ERPs). Indication of focus improved very significantly. This has significant relevance to ADHD [10]. The term healthy may need more definitive diagnosis since this study was published in 1997 just two years after the concept RDS and as such the now developed RDS index was not available.
- Six randomly selected females with a prior history of eating disorders (of which three were also chemically dependent) were contacted at nine months and three years post-treatment with amino-acid precursor and enkephalinase inhibition therapy. All six reported an initial benefit, and while one relapsed at six months, the rest sustained a benefit that exceeded expectations. In addition to this preliminary study, an extended number of 100 former eating disordered patients treated with amino-acid precursor and enkephalinase inhibition therapy resulted in a remarkable 98% reporting significant improvement in both mood and reduced substance craving [11]. While this was not a randomized placebo controlled experiment the number of subjects and the consistency of results seem encouraging and supports other food addiction theories as being common to drug seeking behavior.
- A combination of Trexan [Naltrexone] (a narcotic antagonist) and amino-acids was used to detoxify either methadone or heroin addicts. Results were dramatic regarding significantly enhancing compliance in taking Trexan. When taking Trexan alone for rapid detoxification, the average number of days of compliance calculated on 1000 patients is 37 days.

In this study, 12 subjects received the combined Trexan and amino-acid therapy, taking the combination for an average of 262 days of compliance. This study suggests the importance of coupling amino-acid therapy and enkephalinase inhibition, thereby leading to dopamine balance while blocking the delta-receptors with a pure narcotic antagonist and demonstrating a rapid and novel technique to induce detox in chronic methadone patients and prevent relapse [12]. While this study was rather early in terms of MATS it does point out the adjunctive synergistic intervention of the importance of combining narcotic antagonistic therapy with potential balancing mesolimbic dopamine. As authors of this bibliography type article it is our contention that these findings should be carefully considered in today’s utilization of Naltrexone implant therapy.

- In a first Precision Behavioral Management (PBM®) experiment coupling DNA with a customized formulation, it is relevant to understand that consumption of large quantities of alcohol or carbohydrates (carbohydrate bingeing) stimulates the usage and production of dopamine within the brain. Obesity inpart is due to the need to make up for low dopaminergic activity in the brain’s reward center. This has been given the name of reward deficiency syndrome (RDS) and is used to categorize such biological and genetic influences upon behaviors. RDS needs to be addressed at the same time that behavioral modifications are implemented to treat obese patients adequately. Twenty-four individuals, in a small observational study, completed a survey in which they documented 15 categories of benefit during their experience with an early version of KB220 called GenoTrim, which is a NAAT formulation that is customized to one’s DNA. A statistical analysis of the survey revealed that stress reduction leads to (1) improved sleep, enhanced energy, and improved focus and performance; and (2) reduced appetite, loss of unwanted weight, decreased body inches, and enhanced well-being [13]. The real importance here is that by potentially improving dopaminergic function other benefits realted to a better recovery process from over-eating can be accomplished through Pro-dopamine regulation.
- A one-year prospective study evaluated the effects of taking a KB220 variant called Haveos (Synaptamine)™ on 61 patients in an outpatient program. After 12 weeks in this open-label study, it was found that self-reported craving significantly decreased from program entrance for all 61 patients. Building up to relapse scores showed similar improvement after one year of treatment with significant decreases in stress, depression, anger, anxiety, and drug cravings. Also, there were significant changes in energy level and the ability to refrain from drug-seeking behaviors [14]. The importance here is that the experiment involved a court mandated out-patient residency for these troubled patients in legal jeopardy. The finding of 100% relapse prevention in heroin dependent patients seems surprising but could be due to Federal court mandates.
- In an open clinical study, an Amino-Acid Enkephalinase Inhibition Nutraceutical called Synaptamine (a KB220 variant) improved various symptomatology, including emotional and behavioral symptoms, in 600 alcoholics in recovery. Reductions for cravings, depression, anxiety, anger, fatigue, lack of energy and crisis were all significantly greater than 50%. Specifically, compared to pre and post administration scores, results included reduction of craving, depression, anxiety, anger, fatigue, lack of energy and crises [15]. While this represents a rather large number of patients at a high cost (10K) for the IV therapy which could be avoided by oral dosing potentially as shown in other related studies it does represent further support for the importance of dopamine regulation in alcoholism.
- In the obesity cohort of 122 subjects, the role of Chromium Picolinate (CrP), a major ingredient in KB220, was tested against placebo in groups of obese patients tested for the Taq1 Dopamine D2 Receptor Gene. All 122 subjects were genotyped for the DRD2 A1 or A2 gene. Then this cohort was separated into groups treated with either a placebo (62) or CrP (60). In DRD2 A2 genotype carriers, weight loss and other changes in body composition were significant in this study. Notably, these were not significant for those with the A1/ A1 or A1/A2 allele genotypes. This suggests that the dopaminergic system, specifically the density of the D2 receptors, confers a significant differential therapeutic effect of CrP concerning weight loss and change in body fat. The carriers of DRD2 A1 were found to imbibe in the intake of carbohydrates and fat masking the CrP benefit [16]. This represents one of the first known experiments to incorporate pharmacogenetic testing in terms of a nutrigenomic example in relapse prophylaxis in obesity highlighting the importance of gene polymorphisms and over eating.
- It was hypothesized that genotyping specific known candidate genes would provide DNA-individualized customized nutraceuticals that may have a significant influence on body re-composition by countering various genetic traits. Exploration of the current literature has identified many candidate genes to be associated with obesity. These included the dopamine D2 receptor (DRD2), methylenetetrahydrofolate reductase (MTHFR), serotonin receptor (5-HT2a), Peroxisome Proliferator-Activated Receptor gamma (PPAR-y), and Leptin (OB) genes. In one study, polymorphisms of these five candidate genes were evaluated as important biological targets to be used in the development of a DNA-customized nutraceutical LG839 [DL-phenylalanine, chromium, L-tyrosine other select amino-acids and adaptogens-ingredients in KB220] to combat obesity with particular emphasis on body composition (i.e., BMI). A group of 21 participants was evaluated in a preliminary investigational study of LG839. Pre- and post-ad hoc analysis revealed a significant difference between the starting BMI and the BMI following an average of 41 days (range of 2870 days) of LG839 intake in the 21 individuals. The pre-BMI was 31.2 (weight/height meters^2^) compared to the post-BMI of 30.4 (weight/height meters^2^) with a significance value of p<0.034 (one-tailed). Based on this, it follows that the pre-weight 183.52 compared to the post weight of 179 lb. (p<0.047) was significantly changed. This study also demonstrated trends for reduction of late night snacking, carbohydrate craving reduction, reduction of stress, and reduction of waist circumference. Moreover, in the 41-day period, a trend in weight loss was seen in 71.4% of participants. In this group, 53% lost an average over 2.5% of their starting weight [17]. This experiment is the cornerstone first ever nutrigenomic experiment to couple gene polymorphisms with customized nutrigenomic solutions. This type of genotyping will be in the forefront of treating obesity in the future.
- In a novel experimental DNA-customized nutraceutical study involving 1058 subjects, a subset of 27 people was administered customized LG839 (a KB220 variant). Polymorphic correlates were obtained for many genes (DRD2, LEP, PPAR-gamma2, MTHFR, and 5-HT2A genes) with various clinical parameters. We observed significant results for the appetite suppression; decreases in sugar cravings, snacks and late night eating and even weight. There was also an increase in energy. It is of interest that only the DRD2 gene polymorphism (A1 allele) had a significant Pearson correlation with days on treatment, indicating that carriers of the A1 allele of DRD2 were the most compliant to treatment [18]. The importance of this nutrigenomic and customized Pro-dopamine regulation is that it shows the importance of clinical compliance with the DRD2 A1 allele.
- It was hypothesized that genotyping specific known candidate genes would provide DNA-individualized customized nutraceuticals that may have a significant influence on body composition by countering various genetic traits. Twenty-one subjects were genotyped for the dopamine D2 receptor (DRD2), as well as methylenetetrahydrofolate reductase (MTHFR), the serotonin receptor (5-HT2a), Peroxisome Proliferator Activated Receptor gamma (PPAR-γ), and the Leptin (OB) genes. These genes were systematically evaluated of these five candidate genes as potentially critical biological targets used in the development of the nutraceutical LG839 [DL-phenylalanine, chromium, L-tyrosine other select amino-acids and adaptogens ingredients in KB220] to combat obesity with particular emphasis on body composition (i.e., BMI). In a trail of 41-day, we observed a trend in weight loss whereby 71.4% of subjects lost weight [19]. This experiment represents the third replication of the importance of nutrigenomics in the treatment of obesity.
- Brain dopamine has been referred to as the so-called “anti-stress molecule”. In one study, the anti-anxiety effects of Synaptamine Complex (previous version of KB220), a pro-dopamine regulator, was investigated in a randomized, double-blind placebo-controlled study in alcoholics and polydrug abusers attending an inpatient chemical dependency program. 62 patients receiving Synaptamine Complex had significantly reduced stress compared to patients receiving the placebo. Two-factor measures analyses of variance (ANOVA) divulged significant differences as a function of time and treatment, as well as a significant interaction between them [20]. While these results are supportive of potentially overcoming a hypodopamergia due to stress it does not provide mechanistic evidence of the role of Pro-dopamine regulation and impact on Corticotrophin Releasing Factor (CRF). However, it is known that stress can reduce endogenous opioid peptides in the pituitary and striatum while increasing plasma cortisone.
- A case study evaluated sustained weight loss associated with treatment with the Synaptamine complex was used in conjunction with Diethypropion (Tenuate®), a hormonal repletion therapy, the use of the Rainbow Diet®, and the addition of light exercise. After one year, the 58-year-old male patient’s BMI decreased from 32 to 25.4. Additionally, his body fat composition showed a proportional decrease from 36.91% to 17.8%on the Hologic DEXA scanner. This study represents synergism with known anti-weight loss products [21]. This represents the understanding that obesity represents a multifactorial medical issue whereby induction of putative dopamime homeostasis is necessary.
- In two open-label case studies, intravenous Synaptamine complex (KB220 variant) was administered during protracted abstinence from alcohol and opiates. Results indicate that the qEEG of an alcoholic (with widespread theta activity) and a heroin abuser (with widespread alpha activity) during protracted abstinence are significantly normalized by the administration of one intravenous dose of Synaptamine Complex Variant KB220 [22]. The utilization of neuroimaging especially in opioid use dsorder (OUD) provides imaging evidence related to the neurological effects of Pro-dopamine regulation.
- It is well-known that both food and drug addicts have a “dopamine resistance” due to the Taq A1 allele of the DRD2 receptor gene. Based on earlier studies, the evidence is emerging wherein the potential of utilizing a natural, non-addicting, dopamine precursors may play a significant role in the recovery of individuals with RDS, including those addicted to psychoactive chemicals.

In a randomized, triple-blind, placebo-controlled crossover study with Synaptose Complex KB220Z™, positive outcomes were observed using qEEG. Within the parietal lobes, an increase in both alpha and low beta waves was found. Also, a significant difference between placebo and the compound across the frontal lobes after both weeks one and two was found. The results are divulged a phase change from a low amplitude to a more regulated state by increasing amplitude across the prefrontal cortex. In the first experiment, while 50% of the subjects carried the DRD2 A1 allele, 100% carried at least one risk allele. Specifically, based on our proposed addiction risk score for these 14 participants, 72% of them had a moderate-to-severe addiction risk. Similar findings by repeating the experiment in three additional currently abstinent polydrug abusers carrying the DRD2 A1 allele was also observed [23]. This experiment coupled genetic addiction risk of carrying reduced D2 receptors at birth with Pro-dopamine regulation and benefit displayed by QEEQ.The importance here is the potential regulation of dysregulated prefrontal cortex as obseeved in the cingulate gyrus, and as such this assists in attenuating drug reinstatement or relapse prevention.

- In this study, the effects of combined administration of tyrosine, lecithin, L-glutamine, and L-5-hydroxytryptophan (5-HTP) [ingredients in KB220) on heroin withdrawal symptoms within detoxified heroin addicts was evaluated. In the cluster-randomized placebo-controlled trial, the authors utilized 83 detoxified heroin addicts from a detoxification treatment center in Wuhan, China. The patients in the intervention group (n=41) were given the combined treatment with lecithin, L-glutamine, L-tyrosine, and 5-HTP. Those within the control group (n=42) were administered a placebo. The results showed that scored for withdrawal and insomnia was significantly improved in the intervention group. A more significant reduction in tension-anxiety, depression-dejection, anger-hostility, fatigue-inertia, and total mood disturbance, and a more significant increase in their vigor-activity symptoms were found at day 6 in the intervention group than in the control placebo group [24]. This represents an independent foreign study that validates other studies here in America suggestive of the importance of balancing neurotransmitters in the brain reward circuitry.
- In this study, the authors evaluated a known natural dopaminergic agonist KB220IV (KB220 variant) along with oral variants directed toward overcoming hypodopaminergic function in 129 subjects. In their first pilot experiment, they found a significant reduction of chronic symptoms as measured by the Chronic Abstinence Symptom Severity (CASS) Scale for both intravenous (IV) and oral compared to only oral administration. Specifically, the IV and the oral group did significantly better than the oral only group over the first week and additionally over the following 30 days. In a two year follow-up of 23 subjects who underwent KB220IV therapy (with a minimum of five IV treatments over a one week period) plus orals for at least 30 days: 21 (91%) were sober after six months. Additionally, 19 (82%) had no relapse. Furthermore, 19 (82%) were sober at one year, with 18 (78%) having no relapse. Finally, 21 (91%) were sober at two-years post-treatment with 16 (70%) having no relapse. Overall KB220 variants induced a high degree of abstinence even after a two-year period [25]. While these results dove tail with earlier studies the concept of using IV therapy and the inference of the importance of early IV therapy requires more in-depth research.
- Numerous studies have supported the efficacy of methadone and buprenorphine treatments for the stabilization and maintenance of opioid dependence. As expected, there are often clinically significant withdrawal symptoms that occur upon tapering and cessation of dosage of the opioid.

We present a case study of a 35-year-old Caucasian female. She was prescribed increasing dosages of prescription opioids after carpel tunnel surgery secondary to chronic pain from reflex sympathetic dystrophy and fibromyalgia. Over the next five years, her daily dosage requirements continued to increase to over 80mg of Methadone combined with 300ug/hr Fentanyl transdermal patches, along with combinations of 12–14 1600mcg Actiq lollipop and oral 100mg Morphine and 30mg oxycodone 1–2 tabs q 4–6hr. PRN for any breakthrough pain. Her total monthly prescription costs, including those of supplemental benzodiazepines, hypnotics, and stimulants, exceeded $50k. The patient was genotyped using a reward gene panel including (9 genes 18 alleles): DRD 2,3,4; MOA-A; COMT; DAT1; 5-HTTLPR; OPRM1; and GABRA3. We documented with great care her withdrawal symptoms when she hastily discontinued her buprenorphine/ naloxone combination. At 432 days post-Suboxone® withdrawal, the patient is being maintained on KB220Z, has been urine tested, and is opioid-free. Genotyping data divulged a moderate genetic risk for addiction showing a hypodopaminergic trait [26]. The utilization of an early genetic addiction risk score test displayed a hypodopaminergia in this patient whereby Pro-dopamine regulation for 432 days resulted in opioid free urines and as such prolonged relapse prevention. There are other literature studies showing that D2 agonists also reduce opioid induced withdrawal.

- Lucid dreams might be associated with various psychiatric conditions. This may include Post-Traumatic Stress Disorder (PTSD) and as well as Reward Deficiency Syndrome-associated diagnoses. In a publication, we presented two cases of the dramatic alleviation of terrifying lucid dreams in patients with PTSD. The medication-visit notes revealed changes in the frequency, intensity, and nature of these dreams after the complex putative dopamine agonist KB220Z (a KB220 variant) was added as an adjunct to the first patient’s regimen. When the second PTSD patient, one with a history of lucid nightmares, was administered KB220Z to attenuate his methadone withdrawal symptoms, he incidentally reported dreams full of happiness and laughter [27]. It is conjectured that PTSD is a subset of behaviors tied to RDS. Understanding this and lucid nightmares being linked to hypodopaminergia as explained in this publication [27] it is not surprising that Pro-dopamine regulation had a positive clinical outcome.
- In another publication, we reported eight cases of SUD, with a history of PTSD/RDS and childhood abuse. KB200Z^™^ (KB220 variant), was correlated with the elimination of unpleasant and terrifying, lucid nightmares in 87.5% of our cases. Additionally, one very heavy cocaine abuser had minimal response. Each of the patients was diagnosed with a type of RDS, i.e., ADHD, or Tourette’s syndrome. Furthermore, they also suffered from comorbidities such as Post-Traumatic-Stress-Disorder (PTSD) and various other psychiatric diagnoses as well [28]. While this is supportive of PTSD induced lucid nightmares it extends the work to include ADHD.
- Willuhn *et al.* reported that cocaine users and even non-substance-related addictive behavior increases as the dopaminergic function is decreased. Chronic cocaine use has been shown to cause decreases in D2/ D3 receptors. This also correlated with decreased activation to cues in the cerebellum and occipital lobe in a recent PET study by Volkow’s *et al.* KB220Z (a KB220 variant) induced an increase in BOLD fMRI activation in caudate-accumbens-dopaminergic pathways compared to placebo following 1-hour acute administration in ten abstinent heroin addicts. The authors observed an increase in functional connectivity in a brain network that included medial frontal gyrus, dorsal anterior cingulate, posterior cingulate, nucleus accumbens, cerebellum, and occipital cortex. Results suggested an apparent anti-craving/anti-relapse role of KB220Z in addiction by direct or indirect dopaminergic interaction [29].
- This work extends the positive findings using QEEG with heroin dependence as reported earlier. The BOLD rsfMRI findings showing actuiavtion of dopamine at the caudate-accumbens and the attenuation of the BOLD signal at the cerebellum, provides a clear representation of Pro-dopamine regulation and subsequent induction of “doamine homeostasis”. Importantly, these data supports the role of hypodopaminergia linked to escalation of cocaine seeking behavior.
- In order to explore the initiation of detoxification of addictive patients to opiates/opioids (along with some other anti-withdrawal agents), the authors developed a protocol to be utilized in treatment centers particularly with heavily dependent opiate/opioid subjects. Out of 17 subjects, only three received Buprenorphine/ Naloxone (Bup/nx) along with KB220Z. In this pilot, we first used a dose of KB220Z of 2 oz twice daily before meals along with clonidine and benzodiazepines and other anti-nausea and sleep aids including Gabapentin. The dose of KB220Z was maintained for six days in five individuals. In a second scenario, we utilized a higher dose of 4 oz every 6 hours, over a 6-day period. The higher dose was employed in another 12 patients. It is noteworthy that only three people have relapsed utilizing these two protocols during the first two weeks of the study, allowing for the remaining 82% to be maintained on KB220Z. The patients have been maintained without any additional Bup/nx for a minimum of 120 days and in one subject, 214 days [30]. While this is the first experiment utilizing Pro-dopamine regulation to attenuate opioid induced withdrawal symptoms it does support earlier experiments as noted in this article related to alcohol induced withdrawal and benefits of Pro dopamine regulation in residential treatment.
- A reward dysfunction in dopaminergic/addictive behaviors is supported by a plethora of studies. There is ample evidence that changes in synchronous neural activity between brain regions involved in reward-related behaviors and various cognitive functions, which may significantly contribute to SUDs. One study presented the first evidence showing that a pro-dopaminergic nutraceutical (KB220Z a KB220 variant) significantly enhanced, greater than placebo, the functional connectivity between reward and the cognitive areas of the rat brain. These brain areas included the nucleus accumbens, neighboring hippocampus, anterior cingulate gyrus, anterior thalamic nuclei, prelimbic and infralimbic loci. Results revealed significant functional connectivity with increased brain connectivity and volume recruitment (potentially neuroplasticity), and dopaminergic functionality was found across the brain’s reward circuitry. These increases in functional connectivity tended to be specific to these regions. They were not broadly distributed across the entire brain. While these initial findings have been found within drug naïve rodents, this robust, yet selective response implies clinical relevance for addicted individuals at risk for relapse, who show reductions in functional connectivity after protracted withdrawal. Future studies will evaluate KB220Z in animal models of addiction [31]. While this is a rodent study the findings may have clinical relevance since Eliot Stein’s group at NIDA points out the role of resting state functional connectivity as a prerequisite for all addictive behaviors. The finding here with an increase in resting state functional connectivity provides a mechanism of action related to all the positive clinical benefits observed previously with Pro dopamine regulation. Addionally, the finding of increased brain connectivity and volume recruitment (potentially neuroplasticity) is indeed interesting in light of the role of dopamine in terms of drug seeking prophylaxis.
- In another publication, the authors presented four cases in which there was a noticeable and persistent alleviation of terrifying, lucid nightmares in patients diagnosed with comorbid ADHD and PTSD with a specific SUD (opiate/opioid addictions).

The cessation of these nightmares may be permanent, as the patients had stopped taking the nutraceutical for between 10 −12 months, without recurrence. In case one, the authors evaluated a 47-year-old married man that required the chronic use of Buprenorphine/ Naloxone (Suboxone) treatments. In case two, we had a 32-year-old female with ADHD. In case three, we had a 38-year-old man with ADHD and comorbid SUD. In case four, the authors presented a 50-year-old female with comorbid diagnoses of ADHD, PTSD and alcohol abuse. Perhaps pertinently, to attempt to understand neuroplasticity, they employed KB220Z (a KB220 variant) in non-opioid-addicted rats utilizing fMRI methods. While the authors cannot make any definitive claims because the functional connectivity of the rat brain may not correspond precisely to that of humans, it does provide some interesting clues. The authors did find the following seeding of the dorsal hippocampus, enhanced connectivity volume across several Regions of Interest (ROIs), except for the prefrontal cortex (PFC). Most notably, the PFC is infrequently activated during lucid dreaming [32]. These findings are further supported by the work on rodents and the BOLD activation of dopamine across brain reward circuitry [31].

- With aging, there is a well-known decline in both long and short-term memory. This effect is intensified by epigenetic insults on specific, dopamine-related genes (e.g., DRD2, DAT1). Additionally, long-term memory ability and dopamine function are positively correlated. There is ample evidence that aging is associated with a decrease in brain dopamine D2 receptors, with an acceleration seen in aging-induced dementia. In one study, the authors examined the acute effect of a KB220Z (liquid Nano variant) on a specific aspect of long-term memory performance in a 77-year-old, highly functional male, using the Animal Naming Test (ANT). The improvement of long-term memory retrieval had initially been noted during the patient’s follow-up neurology exam, after he had been, for other reasons, taking KB220z. The patient had been given many ANTs by his primary and, later, another neurologist, from 2013 to 2016. Because the number of ANT observations was small (N=7 with two groups) and the data uncorrelated, a non-parametric Wilcoxon-Mann-Whitney test was performed to test mean differences. After KB220z, the patient had much higher scores (p=0.04762) on the ANT vs. when not taking it. His scores increased from the 30^th^ percentile (pretest) to the 76^th^ percentile, after the first administration of KB220z and, later, to the 98^th^ percentile, after the second administration of KB220z, six months later [33]. Since this is a case study, it requires additional research to support these claims, however, the findings are encouraging. It is well-established that with aging D2 receptors significantly decline along with cognitive ability. These findings of improved memory in an elderly person agree with the known role of dopamine and cognitive ability.
- Attention Deficit-Hyperactivity Disorder (ADHD) often fails to abate beyond adolescence, lasting into adulthood. Recent neuroimaging studies of such individual’s divulged lower baseline of dopamine tone within the brains of these affected individuals might place them at risk for comorbid Substance Use Disorder (SUD) as well. This is an observational case study about the potential for innovative management of Adult ADHD with a non-addictive glutamatergic-dopaminergic optimization complex KB200z (a KB220 variant). Low-resolution electromagnetic tomography (LORETA) was employed to evaluate the effects of KB220z on a 72-year-old male with ADHD, at a baseline condition and subsequently one hour following administration of the compound. Analysis of z-scores that were averaged across various conditions (eyes closed, eyes open and a working memory task), were increased within each frequency band, within the dorsal, anterior, and posterior regions of the cingulate, as well as the right dorsolateral prefrontal cortex during the working memory task with the KB220z compound.

These results are consistent with various human and animal neuroimaging studies that have demonstrated an increased connectivity volume in reward circuitry. This may offer a new approach to ADHD treatment [34]. While a case study the LORETA findings seem clinically relevant but should be met with caution until larger studies could further support this encouraging neuroimaging data.

- The authors report a self-assessment of a highly functional professional under work-related stress following KB220Z (a KB220 variant) use, a liquid (aqua) Nano-glutamatergic-dopaminergic optimization complex (GDOC). The subject took GDOC for one month. During the first three days in the compound, unique brain activation occurred. This resembled that of white noise after 30 minutes, and the sensation was intense for 45 minutes and then dissipated. He described the effect as if his eyesight improved slightly and pointed out that his sense of smell and sleep significantly improved. His subjectively experienced a calming effect that was similar to meditation which could be linked to increased dopamine release. Additionally, he claimed to be able to control going over the edge after a stressful day’s work. This was paired with a slight, yet noticeable increase in energy, increased motivation to work, increased focus and multi-tasking, with a more definite purpose of the tasks at hand. Subjectively, he felt less inhibited in a social setting and suggested that the GDOC increased his Behavioral Activation System (reward) while having a decrease in his Behavioral Inhibition System (caution) [35]. This case study represents the potential role of the glutamatergic-dopaminergic optimization complex (GDOC). As it relates to a highly functional human. The results are not surprising and the suggestion of Pro-dopamine regulation having a duel action by stimulating reward while decrasing inhibition may translate to a better functional social experience asymptotically approaching normalcy.
- Based on previous scientific evidence showing KB220Z nutrigenomic amino-acid therapy (a KB220 variant) to rapidly (post-one-hour) activate dopaminergic pathways in both the pre-frontal cortex cingulate gyrus (relapse loci) and ventral tegmental area-caudate-accumbens-putamen (craving and emotion loci), the patient was prescribed KB220Z. Within one week of utilization, the repetitive paraphilia was eliminated. There were also many other positive effects such as enhanced focus that persisted even after the patient stopped using KB220Z suggesting neuroplasticity (e.g., altruistic thoughts) [36]. This case study is interesting in terms of sexual abarrant behaviors with now known links to hypodopaminergia. There is other unpublished work by our group showing that D2 pharmaceutical agonistic therapy significantly reduces sex addictive behaviors.
- Binge drinking (BD) is a serious but preventable public health problem in the United States and worldwide. BD and substance use disorders may be co-morbid with more generalized Reward Deficiency Syndrome (RDS), characterized by a reduction in dopamine (DA) signaling within the reward pathway, and classically associated with increased drug-seeking behavior. It is postulated here that increasing dopamine availability and thus restoring DA homeostasis in the mesocorticolimbic system could reduce the cravings and motivation to seek and consume ethanol. In this study, the authors used neuro-nutrient KB220, a nutraceutical product designed to supply the brain with molecular precursors and neurotransmitter catabolic inhibitors that augment DA signaling to elucidate its effects specifically on ethanol drinking. Using alcohol-preferring (P) adult male and female rats in an operant binge drinking paradigm genetically, the authors evaluated whether KB220 was sufficient to reduce drinking and the most efficacious route for its administration.

They also evaluated its effects on open field activity and risk-taking behavior. KB220 markedly and immediately reduced binge drinking in both male and female rats via IP and SC administration (p<0.05) whereas P.O. took at least three days to decrease lever pressing in both male and female rats (p<0.05), suggesting a slower metabolism and delayed effect on the brain by P.O. Elevated activity in the open field was significantly decreased (p<0.05), and risk-taking behavior was moderately reduced. Overall, this data suggests that KB220 attenuates drinking behavior and other RDS behaviors in P rats possibly by acting on the dopaminergic system [37]. Work is continuing in this area involving control experiments and other neurochemical experiments to dissect mechanism of action of the Pro-dopamine regulator.

- Over a two-year period, the authors carried out a prospective analysis of 247 outpatients in a very-low-calorie fasting program. Subjects having difficulty attaining their desired weight or maintaining their desired weight constituted the experimental group. At two years, the experimental group that took the amino acid regimen of PhenCal™ (KB220 variant) compared with the non-PhenCal/Centrum™ vitamin control group showed a twofold decrease in the percent overweight (including both males and females). There was a 70% decrease in food cravings for females (63% for males). Additionally, there was a 66% decrease in binge-eating for females (41% for males). Most importantly, the experimental group (PhenCal group) regained only 14.7% of the weight lost during fasting while the control group (non-PhenCal group) regained 41.7% of the lost weight with PhenCal treatment. Furthermore, morbid obesity and binge eating scores were significant predictors of weight gain after two years. In contrast, family history of chemical dependence was most closely associated, although not statistically significant, with improved results with PhenCal. These data suggest that PhenCal may be an anti-obesity adjunct [38]. This represents a very long-term experiment utilizing Pro-doapmine regulation indicating the importance of dopamine balance in obesity. As such emphasizing the importance of dopamine in preventing weight regain in patients. This work begs for a better diagnosis potential genetic risk panel so that a gene guided therapy could potentially reduce risk in terms of weight regain risk by embracing Pro-doapmine regulation.

This published case series demonstrates the epigenetic repair of the Brain Reward Cascade (BRC) by incorporating pro-dopamine regulation with (KB220ZBR) into treatment. Three case studies of patients with various conditions including mitochondrial metabolic disorder, dysautonomia, hyperacusis, unspecified mood and anxiety disorders, chronic fatigue syndrome, and restless leg syndrome are presented. Patients’ responses to treatment of these conditions with the adjunctive chronic administration of a nutraceutical, prodopamine dietary supplement (KB220ZBR), were assessed before supplementation and following chronic treatment. Significant improvements in psychiatric/neurological symptoms were documented. Following confirmatory results, we encourage other neurologists to consider this safe alternative treatment [39] While there is no direct evidence for the role of a Pro-dopamine regulator like KB220 variant on mitochondrial function, based on these new findings, the authors encourage this research.

- This unpublished case study used a double-blind, placebo controlled, cross-over design, to evaluate the effect of KB220Z, a pro-dopamine regulator, on working memory, neuropsychological and behavioral measures of attention in an adult female with Attention Deficit-Hyperactivity Disorder. The participant was tested with eyes closed and with a phonological working memory task. The working memory task required her to remember a random sequence of letters and numbers, and recall them in alphabetical and numerical order.

Response measures included the Conner Continuous Performance Test, the Delis-Kaplan Executive Function Inventory, quantitative electroencephalography (QEEG), as well as Low-resolution Electromagnetic Tomography (LORETA). KB220Z, compared to placebo, improved vigilance, response inhibition and verbal fluency functions. Working memory performance substantially improved in response to KB220Z. Quantitative EEG (QEEG) analysis revealed that absolute power in the alpha and theta EEG bands increased during the working memory task, under KB220Z. In addition, Low-resolution Electromagnetic Tomography analysis revealed increases in current source density at 10Hz in the bilateral dorsal cingulate cortices, bilateral hippocampi and bilateral dorsolateral prefrontal cortices, and at 11Hz in the right hippocampus and right dorsolateral prefrontal cortex, during the working memory task in response to KB220Z. Collectively, these results indicate that pro-dopamine regulation with KB220Z improved working memory and prefrontal, neuropsychological function in conjunction with increased activation of brain regions known to manage executive function, working memory and retrieval of declarative information. These findings replicate and extend our prior case study research with KB220Z and support the value of continued research with this Pro-dopamine regulator [40]. The issue of lack of genetic testing is very relevant and in the future these studies should include genetic addiction risk score (GARS) testing. In fact, these studies would be enhanced greatly when ADHD could be genetically identified.

## Conclusions

As mentioned earlier worldwide daily, millions of people are unable to end their often fatal romance with getting high. For many, the ‘high’ may be just experiencing feelings of normalcy or well-being. The neuroscience community conducts and funds outstanding research using sophisticated neuroimaging and molecular-genetic applied technology to improve understanding of the complex functions of brain reward circuitry, having a key role in addiction symptomatology. While it is an agreed consensus dopamine is a major neurotransmitter implicated in behavioral and substance addictions, there remains controversy about how to modulate dopamine clinically, to treat and prevent various types of addictive disorders (see Table 1.).

As described in this report, a prudent approach may be biphasic, to include a short-term blockade followed by long-term dopaminergic upregulation. The goal of treatment would be to enhance brain reward functional connectivity volume, and target reward deficiency and the stress-like anti reward symptomatology of addiction including attendance of self-help groups [41]. In our studies taking only relapse rates, we find significantly lower relapse rates following KB220 variants in alcohol, opioid, cocaine, and food dependent patient (table 2).

## Figures and Tables

**Figure:**
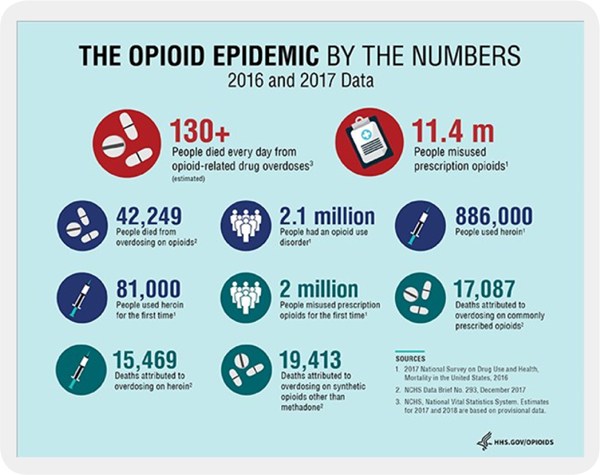
Figure 1

**Table1:** Reward deficiency syndrome behaviors

ADDICTIVE BEHAVIORS		IMPULSIVE BEHAVIORS		OBSESSIVE COMPULSIVE BEHAVIORS	PERSONALITY DISORDERS
Substance Related	Non Substance Related	Spectrum Disorders	Disruptive Impulsive		
Alcohol	Thrill seeking (novelty)	Attention-deficit Hyperactivity	Anti-social	Body Dysmorphic	Paranoid
Cannabis	Sexual Sadism	Tourette and Tic Syndrome	Conduct	Hoarding	Schizoid
Opioids	Sexual Masochism	Autism	Intermittent Explosive	Trichotillo-mania (hair pulling)	Borderline
Sedatives/ Hypnotics	Hypersexual		Oppositional Defiant	Excoriation (skin picking)	Schizotypal
Stimulants	Gambling		Exhibitionistic	Non-suicidal Self-Injury	Histrionic
Tobacco	Internet Gaming				Narcissistic
Glucose					Avoidant
Food					Dependant

* With Permission Blum (2018)

**Table 2:** KB220 Variants and Relapse Rates Compared to Controls in Reward Deficiency Syndrome (RDS)

Patient Category	KB220 Re lapse Rate (N) (%)	Controls Relapse Rate (N) (%)	Reference	Experimental Length of time
Out-Patient Alcohol	15 (26)	15 (87)	Brown *et al*^1^	Ten months
Out-Patient Cocaine	15 (47)	15 (93)	Brown *et al*^1^	Ten months
In-Patient Opioids Detox	29 (18)	NA	Blum *et al*^2^	Four months
Out-patient Alcohol	61 (7)	NA	Chen *et al*^3^	12 months
Out-patient Heroin	4 (0)	NA	Chen *et al*^3^	12 months
Out-patient Alcohol	23 (22)	NA	Miller *et al*^4^	12 months
Out-patient Alcoholics	21 (30)	NA	Miller *et al*^4^	24 months
Out-patient Alcohol	600 (0)	NA	Blum *et al*^5^	3 months**
Out-patient Bariatric	16 (18.2)	11 (82.2)	Blum *et al*^6^	3 months
Out-patient Bariatric	130 (14,7)	117 (41.7)	Blum *et al*.^7^	24 months
Average RDS	91.4 (18.29)	158 (76)*	NA	11.4

Note: Approximately 90% of alcoholics experience at least one relapse in the four years following treatment. Similar relapse rates occur for recovering smokers and heroin addicts, suggesting that many addictive behaviors may share the same behavioral, biochemical, and cognitive components. Like alcoholism, opiate addiction exhibits high relapse rates - the research shows more than 80 percent among those who receive behavioral treatments as a sole treatment. In an important study cited by Reuters, 48 percent of meth users who followed detox with drug rehab were still sober after three months; and 20 percent of those who attended treatment were still abstinent after one year, in contrast to only 7 percent of people who had undergone detox alone or received no treatment.

* While Blum’s group and others have reported on relapse rates using data from NIDA/NIAAA this current number of 76% is well within the relapse percentages seen across the entire scientific literature in the addiction space [8].

** The evaluation took place from 2000 to 2005 so that concerning relapse the total DATA tabulated from an extensive database and each subject’s duration of relapse was grouped. However, it is noted that the minimum criteria to enter into the study was a receipt of an at least 3-month supply of oral KB220 and as such, we considered only to be three months a very truncated effect.

## Abbreviations

5-HT2a: specific serotonin receptor
5-HTP: L-5-hydroxytrytophan
5-HTLPR: genetic variant of serotonin transporter gene
A1: specific genotype of the D2 dopamine receptor
A2: specific genotype of the D2 dopamine receptor
ADD: attention-deficit disorder
ADHD: attention-deficit/hyperactivity disorder
AMA: against medical advice
ANOVA: analysis of the variance
ANT: Animal Naming Test
BD: binge drinking
BMI: body mass index
BOLD: blood oxygen level dependent (functional magnetic resonance imaging sequence)
BRC: Brain Reward Cascade
Bup/nx: Buprenorphine/Naloxone drug combination
C: no supplement control
CASS: Chronic Abstinence Symptom Severity
CDC: Centers for Disease Control and Prevention
CI: confidence interval
COMT: Catechol-O-methyltransferase
CrP: Chromium Picolinate
D2: specific dopamine receptor
D3: specific dopamine receptor
DA: dopamine
DAT-1: dopamine transporter polymorphism
DEXA: Dual Energy X-Ray Absorptiometry
DNA: Deoxyribonucleic acid
DRD: dopamine receptor genes
DRD2: dopamine D2 receptor
DUI: driving under the influence
ERPs: event-related potentials
FDA: Food and Drug Administration
fMRI: functional magnetic resonance imaging
GABRA3: gene that encodes Gamma-aminobutyric acid receptor subunit alpha-3
GARS®: Genetic Addiction Risk Score
GDOC: glutamatergic-dopaminergic optimization complex
GenoTrim: early variant of restoregen
hrs.: hours
IP: Intraperitoneal injection
IV: intravenous
Kb220: Neuro-nutrient variant
KB220IV: Neuro-nutrient variant
KB220ZTM: Neuro-nutrient variant
lb. or lbs.: weight in pounds
LEP: Leptin gene (see OB)
LG839: restoregen variant
LORETA: Low-resolution electromagnetic tomography
MATS: medication-assisted treatments
mcg: microgram
mg: milligram
MOA-A: Monoamine oxidase type A
MTHFR: methylenetetrahydrofolate reductase gene
mV: millivolts
NAATTM: Neuroadaptagen Amino-Acid Therapy
NIAAA: The National Institute on Alcohol Abuse and Alcoholism
NIDA: The National Institute on Drug Abuse
OB: obesity genotype for Leptin
OPRM1: protein encoding for Opioid Receptor Mu 1
oz: ounce
P300: event-related potential component (either the third or the one occurring 300 ms. poststimulus related to decision making
p: probability score
P: preferring (referring to a specific substance)
P.O.: Per os (oral administration of medication)
PAM®: Precision Addiction Management
PBM: Precision Behavioral Management
PCAL-103: experimental neuronutrient (precursor amino acid loading and enkephalinase inhibition)
PET: Positron emission tomography
PFC: prefrontal cortex
PhenCal: KB220 variant
PPAR-γ: Peroxisome Proliferator-Activated Receptor gamma
PRN: pro re nata (as needed)
PTSD: Post-Traumatic Stress Disorder
q: every
qEEG: quantitative electroencephalography
RDS: Reward Deficiency Syndrome
ROIs: regions of interest
RSS: Reward Surfeit Syndrome
SC: subcutaneous
SUD: Substance Use Disorder
T: Tropamine
t: t-score from a t-test
